# Health insurance mediation of the Mexican American non-Hispanic white disparity on early breast cancer diagnosis

**DOI:** 10.1186/2193-1801-2-285

**Published:** 2013-06-28

**Authors:** Sundus Haji-Jama, Kevin M Gorey, Isaac N Luginaah, Madhan K Balagurusamy, Caroline Hamm

**Affiliations:** School of Social Work, University of Windsor, 401 Sunset Avenue, Windsor, Ontario N9B 3P4 Canada; Department of Geography, University of Western Ontario, London, Ontario Canada; Windsor Regional Cancer Center, Windsor, Ontario Canada; Department of Medicine, Division of General Internal Medicine, School of Medicine and Dentistry, University of Western Ontario, London, Ontario Canada

**Keywords:** Mexican American, Barrio, Gateway neighborhood, Poverty, Health insurance, Breast cancer, Early diagnosis

## Abstract

We examined health insurance mediation of the Mexican American (MA) non-Hispanic white (NHW) disparity on early breast cancer diagnosis. Based on social capital and barrio advantage theories, we hypothesized a 3-way ethnicity by poverty by health insurance interaction, that is, that 2-way poverty by health insurance interaction effects would differ between ethnic groups. We secondarily analyzed registry data for 303 MA and 3,611 NHW women diagnosed with breast cancer between 1996 and 2000 who were originally followed until 2011. Predictors of early, node negative (NN) disease at diagnosis were analyzed. Socioeconomic data were obtained from the 2000 census to categorize neighborhood poverty: high (30% or more of the census tract households were poor), middle (5% to 29% poor) and low (less than 5% poor). Barrios were neighborhoods where 50% or more of the residents were MA. Primary health insurers were Medicaid, Medicare, private or none. MA women were 13% less likely to be diagnosed early with NN disease (RR = 0.87), but this MA-NHW disparity was completely mediated by the main and interacting effects of health insurance. Advantages of health insurance were largest in low poverty neighborhoods among NHW women (RR = 1.20) while among MA women they were, paradoxically, largest in high poverty, MA barrios (RR = 1.45). Advantages of being privately insured were observed for all. Medicare seemed additionally instrumental for NHW women and Medicaid for MA women. These findings are consistent with the theory that more facilitative social and economic capital is available to MA women in barrios and to NHW women in more affluent neighborhoods. It is there that each respective group of women is probably best able to absorb the indirect and direct, but uncovered, costs of breast cancer screening and diagnosis.

## Background

During what has come to be known as the Great Recession, the census bureau estimated that the prevalence of poverty had increased nearly 25% in America over only four years (from 37.5 million in 2007 to 46.2 million in 2011). During the same era the prevalence of Americans without health insurance increased by more than 10% to 50 million (DeNavas-Walt et al. 2012). But if the underinsured were included, the estimate doubled to 100 million or approximately one of every three Americans (Kaiser Family Foundation 2012). When viewed through an ethnic lens, such inequities are even more startling. For example, the prevalence of being uninsured among Hispanic Americans (32.4%) was estimated to be nearly three times greater than among non-Hispanic white (NHW) Americans in 2009 (12.0% (Kaplan & Inguanzo 2011)). And among Hispanics, the prevalence was highest among Mexican Americans (MA), four of every ten of them lacking any form of health insurance, public or private (Miranda et al. 2011).

Even in the decade that preceded the great recession, the social risks that arose out of being uninsured and poor were stronger in some places and among some people. In California, the state with the largest MA population, the concentration of poor people into extremely poor neighborhoods rose substantially. And among Hispanic people, MA women in particular, the concentrations of the poorest poor and the least insured into so-called barrios were dramatic (Acosta 2010; Berube & Frey 2005; Jargowsky 2005). Our research group has been analyzing the effects of these social forces on cancer care in California over the past 15 years (Gorey et al. 2011; Gorey et al. 2009). This study aims to connect this work to the burgeoning social capital-based theoretical explanations for the Hispanic paradox. Diverse health benefits seem to be enjoyed by otherwise quite socioeconomically vulnerable people who live in Hispanic enclaves (Keegan et al. 2010; Mair et al. 2010; Cagney et al. 2007), especially in barrio neighborhoods that are predominantly populated by first generation immigrants from Mexico (Osypuk et al. 2010). When discussing healthcare disparities that Hispanics might face, it is important to analyze different Hispanic groups separately and avoid the risk of missing important clinical and social differences. Different groups may face different obstacles to accessing healthcare resources (Miranda et al. 2011).

Focusing on the cancer care and survival experiences of extremely poor people with cancers of great public health and human significance—breast and colon cancer—we have consistently observed that health insurance does not only matter, but indeed is critical. Adequate health insurance, be it private or public, seems to be strongly associated with access to the best available treatments and outcomes for all Americans. But for women, particularly women with the most treatable types of cancer such as localized, node negative (NN) breast cancer, health insurance seems to all but completely mediate or buffer the profoundly disadvantaging effects of poverty. We also observed two distinct, but theoretically related interactions of poverty and health insurance. First, the advantaging effects of health insurance were much stronger in low poverty neighborhoods, where less than five percent of the households were poor, than in high poverty neighborhoods, where thirty percent or more of the households were poor (Gorey et al. 2013; Gorey et al. 2012). Such high poverty neighborhoods have been described as places of prevalent demographic vulnerability that are particularly distressed for their lack of social and economic capital (Wilson 2012; Jargowsky 1997; Jargowsky & Bane 1991). It appears that women with breast cancer in more affluent neighborhoods, where more facilitative social and economic capital is available, are probably better able to absorb the indirect and direct, but uncovered, costs of care. Second, among MA women, the advantaging effects of health insurance were particularly strong in a certain type of high poverty neighborhood, that is, in barrios where the majority of the residents were MA (KMG, unpublished observations). Though seemingly paradoxical, such findings are consistent with the theory that MA barrios, even though they tend to be places of high poverty, may provide their residents with relatively more instrumental social and economic supports (Aranda et al. 2011; Markides & Eschbach 2005; Eschbach et al. 2004; Suarez 1994; Markides & Coreil 1986). This demonstrates that the effects of health insurance do not operate in a social vacuum. Health insurance surely matters, but so too does place and culture.

It seems that the interacting effects of being uninsured or underinsured, being poor and being an ethnic minority woman of color have been rather well studied during the post-diagnostic phase of breast cancer care. Much less is known about the diagnostic phase of care even though a number of studies have suggested that having adequate health insurance coverage at least partially mediates poverty and MA screening disadvantages (Miranda et al. 2011; Garcia et al. 2012; Gonzalez et al. 2012). It has already been established in this context that MA women are much less likely than NHW women to be diagnosed relatively early with NN breast cancer (KMG, unpublished observations), a type of breast cancer that has not yet spread to any regional lymph nodes and so typically has an excellent prognosis. Aiming to advance theoretical and practical understandings about this ethnic diagnostic gap, we advanced these hypotheses. First, the MA-NHW diagnostic gap is mediated by health insurance. Second, among NHW women the health insurance-early diagnosis relationship is moderated by poverty such that health insurance is less effective in high poverty neighborhoods. And third, among MA women in high poverty neighborhoods, the health insurance-early diagnosis relationship is moderated by barrio status such that health insurance is more effective in MA barrios.

## Methods

The sampling frame was the California cancer registry. Study participants were originally randomly selected from three geographic and three socioeconomic place strata. Geographic strata were very large metropolitan areas (San Diego, San Francisco and Los Angeles), smaller metropolitan areas (Salinas, Modesto, Stockton, Bakersfield and Fresno) and rural places. Socioeconomic strata were based on the prevalence of poor households in census tract-defined neighborhoods: high poverty (30% or more), middle poverty (5% to 29%) and low poverty (less than 5% poor (Census Bureau 2002)). Data was obtained for 303 MA and 3,611 NHW women with breast cancer, diagnosed and staged between 1996 and 2000, who were originally followed until 2011. Most of the MA women were identified directly through medical records (77.8%), the remainder through a validated algorithm using Hispanic surnames and maiden names, birthplace, race and other record linkages (sensitivity = 84.4% and specificity = 99.1% (NAACCR Race and Ethnicity Work Group 2009)). MA barrios were defined as neighborhoods where 50% or more of the residents were MA in 2000. We explored other barrio criteria from 33% to 75% MA. The 50% criterion had the most predictive validity for these analyses. Health insurance and breast cancer care variables were extracted from hospital and physician office charts and clinic reports.

We used logistic regression models to test hypotheses about the mediating and moderating effects of poverty and health insurers in predicting binary (node negative or positive) breast cancer stage at diagnosis. Odds ratios (OR) and 95% confidence intervals (CI) were estimated. We also provided practical assessments more germane to clinical or policy significance. All rates were directly adjusted for age and tumor grade using this study’s sample as the standard. Then we used standardized rate ratios (RR) for all between-group comparisons with pooled 95% CIs. Statistical model tests are presented in tables. Practical significance indices are presented in the text. Other methodological details have been reported (Gorey et al. 2013; Gorey et al. 2012).

## Results

### Description of samples

Descriptive characteristics of the MA and NHW samples of women with breast cancer are displayed in Table 1. All of the unadjusted, statistically significant, comparisons seemed quite consistent with existing knowledge. MA women, approximately nine of every ten of whom were first generation immigrants, were much more likely to live in high poverty, large urban neighborhood barrios and to be either uninsured or insured by Medicaid. Furthermore, annual household incomes among MAs were much lower than NHWs (median income of $26,000 versus $52,225, median test *p* < .05). Typically being more than a decade younger at diagnosis than their NHW counterparts (median age of 49.5 versus 62.5, median test *p* < .05), the MA women were more likely to have never been married and less likely to be widowed. Finally, breast tumors among the MA women were more advanced and less well differentiated.

**Table 1:** **Place, socioeconomic, demographic and tumor descriptive profiles: Mexican American and non-Hispanic white women**

Variable	Mexican American^a^		Non-Hispanic white	
	Sample	Percentage	Sample	Percentage
Geographic place^*^				
Large urban	130	42.9	1,065	29.5
Smaller urban	85	28.1	1,278	35.4
Rural	88	29.0	1,268	35.1
Neighborhood poverty prevalence,^*^ %				
< 5	28	9.2	1,525	42.2
5-29	77	25.4	1,300	36.0
≥ 30	198	65.3	786	21.8
Neighborhood Mexican American prevalence,^*^ %				
< 25	50	16.5	2,721	75.4
25-49	83	27.4	617	17.1
≥ 50	170	56.1	273	7.6
Primary health insurer^*^				
Uninsured	61	20.1	299	8.3
Medicaid	74	24.4	134	3.7
Medicare	54	17.8	1,082	30.0
Private	114	37.6	2,096	58.0
Age at diagnosis,^*^ y				
25-44	98	32.3	411	11.4
45-54	78	25.7	762	21.1
55-64	66	21.8	768	21.3
≥ 65	61	20.2	1,670	46.2
Marital status^*^				
Married	178	59.9	2,002	56.6
Never married	52	17.5	369	10.4
Separated or divorced	28	9.5	467	13.2
Widowed	39	13.1	700	19.8
Histological grade^*^				
I, well differentiated	38	12.5	891	24.7
II, moderately	110	36.3	1,556	43.1
III or IV, poorly differentiated	155	51.2	1,164	32.2
Summary stage^*^				
Local-regional, node negative	169	55.8	2,485	68.8
Local-regional, node positive	126	41.6	1,013	28.1
Distally metastasized	8	2.6	113	3.1

^a^ Most (88%) were first generation immigrants born in Mexico and this characteristic did not differ significantly between barrio and non-barrio residents.

^*^p < .05 for between-ethnic group difference (*χ*^2^ test).

### Mediation and moderation of the ethnicity-early breast cancer diagnosis relationship

Significant, otherwise unadjusted, age- and grade-adjusted effects of ethnicity, poverty and primary health insurer on early diagnosis of NN breast cancer are displayed in the top of Table 2. Moving down the table to the fully adjusted regression model, the apparent effect of being MA (OR = 0.77; 95% CI = 0.60, 0.99 [RR = 0.87; 95% CI = 0.80, 0.94]) was no longer significant in the presence of health insurance and poverty (OR = 1.12; 95% CI = 0.77, 1.63). In fact, when poverty was removed from the model the MA effect remained null (OR = 1.08; 95% CI = 0.76, 1.54, data not shown). Having private health insurance or Medicare coverage seemed to completely mediate the MA-NHW disparity on early breast cancer diagnosis. The mechanism of such mediation seems to be through a rather complex 3-way interaction of ethnicity, poverty and health insurance. This effect moderation, meaning essentially that the effect of the 2-way poverty by health insurance interaction differs by ethnicity, is depicted in Table 3.

**Table 2:** **Logistic regression main effects and interactions of ethnicity, neighborhood poverty and primary health insurers on early diagnosis of node negative breast cancer**

Predictor variables (Baseline Comparison)	Odds ratio	95 percent confidence interval
Separate main effect models		
Ethnicity (Non-Hispanic white)		
Mexican American	**0.77**	0.60, 0.99
Neighborhood poverty (low poverty)		
Middle poverty	0.92	0.78, 1.08
High poverty	**0.79**	0.66, 0.94
Primary health insurer (uninsured or Medicaid)		
Private or Medicare	**1.38**	1.14, 1.67
Full model		
Ethnicity (Non-Hispanic white)		
Mexican American	1.12	0.77, 1.63
Neighborhood poverty (low poverty)		
Middle poverty	0.95	0.80, 1.12
High poverty	**0.86** ^*^	0.71, 1.03
Primary health insurer (uninsured or Medicaid)		
Private or Medicare	**1.40**	1.13, 1.73
Ethnicity by neighborhood poverty by primary health insurer	**0.64** ^*^	0.39, 1.07

Notes: All effects were age and grade-adjusted. After these covariates, ethnicity, poverty, health insurance and their interactions were accounted for, geographic place and marital status did not enter the full model. Barrio was not entered as it was not theoretically or hypothetically meaningful for NHW women.

^*^p < .10.

**Table 3:** **Logistic regression main effects and interactions of neighborhood poverty and primary health insurer on early diagnosis of node negative breast cancer for Mexican American and non-Hispanic white women**

Predictor Variables		Mexican American				Non-Hispanic White			
		Sample	OR	(95% CI)		Sample		OR	(95% CI)
Neighborhood poverty									
< 5% poor		28	1.00			1,525		1.00	
5-29% poor		77	1.07	(0.40, 2.89)		1,300		0.90	(0.76, 1.07)
> 30% poor		198	0.66	(0.26, 1.69)		786		0.84	(0.68, 1.04)
Neighborhood Mexican American prevalence									
< 50%		133	1.00			3,338		1.00	
> 50% (Barrio)		170	0.68	(0.36, 1.27)		273		0.99	(0.74, 1.33)
Primary health insurer									
Uninsured or underinsured^a^		115	1.00			433		1.00	
Adequately insured^b^		188	0.87	(0.44, 1.73)		3,178		**1.72**	(1.31, 2.27)
		Poverty by barrio by insurer				Poverty by insurer			
Significant interactions		303	**2.33** ^*^	(0.94, 5.82)		3,611		**1.61**	(1.07, 2.42)
Poverty by insurer interaction for non-Hispanic white women									
		< 5% poor				> 5% poor			
Predictor Variables		Sample	OR	(95% CI)		Sample		OR	(95% CI)
Primary health insurer									
Uninsured or Medicaid		129	1.00			304		1.00	
Private or Medicare		1,396	**1.75**	(1.20, 2.57)		1,782		**1.29** ^*^	(0.99, 1.67)
Poverty by barrio by insurer interaction for Mexican American women									
				> 30% poor					
	< 30% poor			< 50% Mexican American			> 50% Mexican American		
	Sample	OR	(95% CI)	Sample	OR	(95% CI)	Sample	OR	(95% CI)
Primary health insurer									
Uninsured/Medicare	30	1.00		32	1.00		53	1.00	
Private/Medicaid	75	1.45	(0.58, 3.60)	33	0.69	(0.22, 2.11)	80	**2.09** ^*^	(0.96, 4.58)

Notes: All effects were adjusted for age, grade and all other main and interaction effects. Adjustment of the MA regression model for birthplace made no practical difference in findings.

^a^ Uninsured or Medicare for MA women and uninsured or Medicaid for NHW women.

^b^ Private insurance or Medicaid for MA women and private insurance or Medicare for NHW women.

^*^p < .10.

Separate MA and NHW regression models are displayed in the table. Of first note is the lack of main effects in the presence of significant interactions. Only the main effect of health insurance was significant for NHW women (OR = 1.72, right column), the Medicare or privately insured (69.5%) being 13% more likely to be diagnosed with NN disease than were the Medicaid or uninsured (61.5%, RR = 1.13; 95% CI = 1.05, 1.21). The poverty by health insurer interaction depicted near the bottom of the table indicates, as hypothesized, that the advantaging effect of having adequate health insurance, Medicare or private, was larger in low poverty neighborhoods (OR = 1.75) than in middle to high poverty neighborhoods (OR = 1.29) for NHW women. As for practical significance, the size of the adequate insurance-early diagnosis effect in low poverty neighborhoods (respective early diagnosis rates among adequately and inadequately insured of 70.8% vs. 59.0%, RR = 1.20; 95% CI = 1.06, 1.36) was nearly twice the size of the effect in higher poverty neighborhoods (68.5% vs. 61.4%, RR = 1.12; 95% CI = 1.02, 1.23).

The poverty by barrio by health insurer interaction among MA women is depicted at the bottom of the table. As hypothesized, the advantaging effect of having adequate health insurance, Medicaid or private, was largest in MA barrios even though they were all also high poverty neighborhoods. In fact, these were the only places were health insurance seemed to have a significant protective effect (OR = 2.09) for MA women. And in a practical sense, the effect was quite large. In barrios, the Medicaid or privately insured (73.9%) were 45% more likely to be diagnosed with NN disease than were the uninsured or those covered by Medicare (50.8%, RR = 1.45; 95% CI = 1.11, 1.89). Furthermore, such adequately insured barrio residents (73.9%) even seemed to enjoy early diagnoses at a rate on par with similarly well insured NHW women who lived in relatively affluent neighborhoods (70.8%, RR = 1.04; 95% CI = 0.91, 1.19). Finally, we noted that MA barrio residents were more likely to be married (63.9%) and typically had slightly higher annual household incomes (median = $24,600) than MAs who lived in similarly high poverty, but non-barrio neighborhoods (44.6% and $22,525, both *χ*^2^ and median tests, *p* < .05). There was also a non-significant trend for more barrio residents to have adequate health insurance coverage (60.2% vs. 50.8%).

## Discussion

MA women with breast cancer were much less likely to have been diagnosed relatively early, before their disease had spread to regional lymph nodes, but this MA-NHW disparity was completely mediated by the main and interacting effects of health insurance. Advantages of health insurance were largest in low poverty neighborhoods for NHW women, while among MA women they were, paradoxically, largest in high poverty MA barrios. In fact, the highest rate of early NN breast cancer diagnosis was among such MA women with adequate health insurance who lived in MA barrios, with three-quarters having NN disease at the time of their diagnosis. Consistent advantages of being privately insured were also observed for all study participants, MA and NHW, with Medicare coverage seemingly more instrumental for the older cohort of NHW women and Medicaid coverage more so for the poorer cohort of MA women.

It seems that the effectiveness of public and private health insurance programs is significantly impacted by the availability of other key resources. In more well-to-do neighborhoods where social and economic capital abound most NHW women with breast cancer seem quite able to absorb the indirect and additional uncovered, direct costs of care. High poverty neighborhoods on the other hand, with their relative lack of such capital reserves, seem to remain as described more than a generation ago by William Julius Wilson, places of “true disadvantage” (Wilson 2012), especially for the women who live there. Not only are they much more likely to be uninsured or underinsured (Gorey et al. 2013; Gorey et al. 2012), but even when publicly or privately insured, these programs seem much less effective there than they are in places of lower poverty. Seemingly paradoxically, even within high poverty neighborhoods, a very strong advantaging effect of having health insurance was observed among MA women who lived in barrios where the majority of their neighbors were MA. These findings in support of the “barrio advantage” theory, suggest that adequate health insurance, in concert with other social and economic resources that may be more available in largely MA neighborhoods likely potentiate each other. It stands to reason that having the additional capital of either private or public health insurance could operate to potentiate the strengths and resiliencies that already seem to exist in barrios.

### Gateway Mexican American neighborhoods

By purposefully oversampling women with breast cancer from some of the poorest neighborhoods in California, it seems that we also oversampled recent immigrants among our MA subsample. In fact, nine of every ten of the MA women in our study were first generation immigrants. The extremely low-income barrios we studied were comprised typically (70%) of MAs who were also almost exclusively (90%) first generation immigrants. High immigrant “gateway” Hispanic neighborhoods in Los Angeles were recently validated through mixed-methods by the geographer Regan Maas (Maas 2011). Her nuanced analyses found much support for the notion that it is such “first point of contact” places where countries of origin, for example, Mexican cultural norms, are probably strongest and so social capital is strongest and most supportive. The MA barrios we studied were consistent with Maas’ gateway neighborhood criteria (prevalent low-income and high-immigrant populations) as it seems was the specificity of the health protective effects we observed. Both general health benefits that she observed in Los Angeles and the cancer diagnostic advantages that we observed across California were restricted to low-income, high-immigrant gateway neighborhoods. Furthermore, Maas’ qualitative findings of “tight knit, close mutigenerational social networks of family members” that seem most strongly associated with practical economic, health and even health care benefits in gateway neighborhoods are consistent with a generation of sociological theorizing that seem a very good fit with our findings on MA women with breast cancer (Portes & Bach 1985; Palloni & Morenoff 2001; Haas et al. 2004).

Our finding of earlier breast cancer diagnosis among MA women who resided in MA barrios or gateway neighborhoods was inconsistent with two previous studies that found later diagnoses in Hispanic enclaves (Keegan et al. 2010; Reyes-Ortiz et al. 2008). Those other studies, however, studied more ethnically diverse Hispanic women. Likely of more importance is that they studied somewhat higher income neighborhoods that included substantially more second, third and even fourth generation immigrants. Maas also described such neighborhoods and confirmed that they do not seem to offer the same sorts of bonding social capital or health protections that gateway neighborhoods do. She theorized that later, more acculturated, immigrant cohorts have weaker connections to cultural traditions and so probably offer each other increasingly less instrumental social support.

### Potential limitation

We think that some of our analyses were statistically powerful, especially the ethnicity-health insurance-early diagnosis mediation hypothesis test that analyzed the experiences of nearly 4,000 women. That analysis provided rather precise effect estimates that may engender substantial confidence. Admittedly, certain moderator hypotheses we examined, especially those related to the increasingly specific experiences of the nearly 200 MA women in high poverty neighborhoods and who lived in MA barrios (*n* = 133) and were adequately or inadequately insured (respective samples of 80 and 53) were increasingly exploratory. Also, this observational study did not provide the direct means of making causal inferences. However, we think that its findings are consistent with well-established causal criteria. For example, they seem quite theoretically plausible in that they are consistent with much extant sociological theory and they seem consistent with much research that has been accomplished across diverse geographic and methodological contexts. We hope that researchers with access to national data will advance confidence in this field’s knowledge by systematically replicating these analyses.

This study has a key strength as well. In focusing on diagnosis we think that we effectively ruled out the most prevalent confound explanations for Hispanic-paradoxical or barrio mortality advantages. First, through mathematical modeling we essentially matched MA and NHW women on two proxies of disease virulence: age and tumor grade. Therefore, the two analytic groups were similarly diseased or relatively health, making the healthy immigrant alternative explanations unlikely. Second, the fact that we observed MA barrio advantages during the initial phase of diagnostic breast cancer care probably also effectively ruled out return migration or so-called “salmon bias” as well as other selective mortality explanations.

## Conclusion

These findings reaffirm the preventive impact of health insurance especially among those at greatest risk of not having adequate coverage. They are also consistent with the theory that more facilitative social and economic capital is available to MA women in barrios and to NHW women in more affluent neighborhoods. It is there that each respective group of women with breast cancer is probably best able to absorb the indirect and direct, but uncovered, costs of care. Policy makers need to understand that even covered health care presently comes with myriad of costs. And while many seem able to absorb them, many others do not.

### Ethical standards

This study was reviewed and cleared by the University of Windsor research ethics board.
